# Correction: Performance of Generative Pretrained Transformer on the National Medical Licensing Examination in Japan

**DOI:** 10.1371/journal.pdig.0001682

**Published:** 2026-08-21

**Authors:** Yudai Tanaka, Takuto Nakata, Ko Aiga, Takahide Etani, Ryota Muramatsu, Shun Katagiri, Hiroyuki Kawai, Fumiya Higashino, Masahiro Enomoto, Masao Noda, Mitsuhiro Kometani, Masayuki Takamura, Takashi Yoneda, Hiroaki Kakizaki, Akihiro Nomura

In the Improving performance through English translation and optimized prompts in 116^th^ NMLE (2022) subsection of the Results, there is an error in the second and fourth sentences of the second paragraph. The correct second sentence is: Initially, we obtained the scoring rates of 52.8% (85/161 points) for essential questions and 53.2% (109/205 points) for basic and clinical questions with an output error rate of 5.5%. The correct fourth sentence is: Although this marginally increased the scoring rates to 61.5% (99/161) for essential questions and 54.6% (112/205) for basic and clinical questions, the output errors increased to 15.5% (Fig 2).

In the Improving performance through English translation and optimized prompts in 116^th^ NMLE (2022) subsection of the Results, there is an error in the third to sixth sentences of the third paragraph. The correct sentences are: These optimized prompts improved the scoring rates to 65.8% (106/161) for essential questions and 62.9% (129/205) for basic and clinical questions with a reduced output error rate of 4.1%. Additionally, we applied the above-optimized prompts to the GPT-4, which demonstrated the scoring rates of 88.8% (143/161) for essential questions and 81.5% (167/205) for basic and clinical questions and a minimal error rate of 0.3% (Fig 2). Furthermore, we applied the GPT-4 model with optimized prompts to the entire set of 394 questions (text-only) in the 116th NMLE, regardless of containing image data (originally 400 questions, but six were officially removed from the scoring evaluation). This optimal model also showed a scoring rate of 88.7% (173/195) for essential questions and 78.1% (232/297) for basic and clinical questions, whose rates were higher than the minimum passing rates as well as those previously reported state-of-the-art model [18].

In the Discussion, there is an error in the second sentence of the second paragraph. The correct sentence is: Although GPT-3.5 achieved a scoring rate of 52.8% for essential questions and 53.2% for basic and clinical questions for Japanese questions, it increased to 61.5% for essential questions and 54.6% for basic and clinical questions after translating the questions into English.

In the Discussion, there is an error in the fifth sentence of the second paragraph. The correct sentence is: In particular, after adjusting our prompts to include a translation procedure into plain English and modifying the output format based on the question type, the scoring rates increased to 65.8% for essential questions and 62.9% for basic and clinical questions.

In the Discussion, there is an error in the eighth sentence of the second paragraph. The correct sentence is: Although the error rate increased to 15.5% upon translating the Japanese questions into English, it notably decreased to 4.1% after adjusting the prompts by including the format of the output.

In the Discussion, there is an error in the 11^th^ sentence of the second paragraph. The correct sentence is: Finally, upon applying these optimized prompts to GPT-4, the scoring rates increased to 88.8% for essential questions and 81.5% for basic and clinical questions with higher scoring rates than the previously reported state-of-the-art model prompt, and the error rate plummeted to 1.1%.

In Fig 2, there is an error in the reported scores for the 116th National Medical Licensing Examination (NMLE). Please see the correct Fig 2 here.

**Fig 2:**
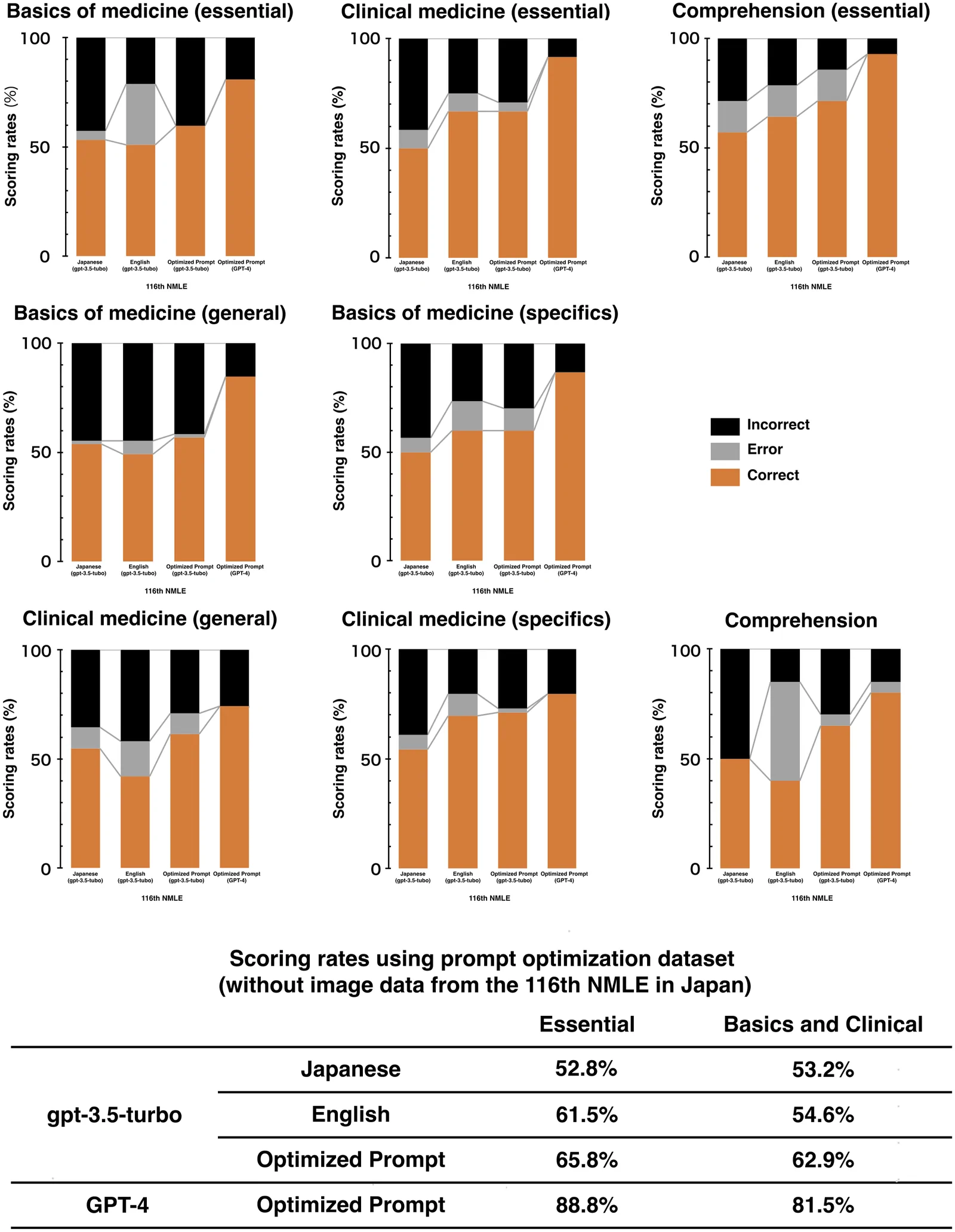
Variations in the scoring rates across languages, prompt adjusting levels, and GPT models. Translating the Japanese questions into English text improved the scoring rates; however, it increased the output error rate. Upon further adjusting the prompts, the scoring rates improved, and the output error decreased. Moreover, switching from the GPT-3.5 model to the GPT-4 model enhanced the scoring rates and almost eliminated errors.
