# Supplementary material for: Performance of Generative Pretrained Transformer on the National Medical Licensing Examination in Japan
Source: PLOS Digit Health. 2024 Jan 23;3(1):e0000433. doi: 10.1371/journal.pdig.0000433 (PMC10805303; doi:10.1371/journal.pdig.0000433)
Supplement: Attachment — Submitted filename: Response to Reviewers FINAL.pdf [file pdig.0000433.s002.pdf]

## Response to Reviewers for PONE-D-23-04728R2

## "Performance of Generative Pretrained Transformer on the National Medical Licensing Examination in Japan"

We would like to thank the editor and the reviewers for their helpful and constructive comments. Their suggestions are greatly appreciated, and we have carefully revised the manuscript. Our responses are listed below, point-by-point.

**Response to Reviewer #1**

**-Major comments-**

**1.** *One of the most serious concerns about this work is the difference between this work and Kasai et al. (2023). I understand that this submission is contemporary to and independent from theirs, but since they published it before your submission, at least the authors need to credit their work and explain the difference between their work and this work. As far as I understand correctly, the finding about the correct answer rates for GPT-3.5 and GPT-4 is pretty similar to their finding (I'm not saying this diminishes the value of this work because this work presents several prompts and reports detailed analysis), so the novelty of this work should be stated more explicitly (compared to theirs).*

**Author reply:** We appreciate your suggestion. As you pointed out, Kasai et al. reported the similar performance as our findings in terms of correct answer rates using GPT-3.5 or GPT-4 based ChatGPT for the latest 117$^{th}$ (2023) National Medical Exam in Japan (NLME). However, since they have not shown any detailed prompts they used for the analyses in the preprint manuscript (even for their version 2 paper), we could not replicate or confirmed validity of any of their results. Thus, in addition to just reporting the performance of ChatGPT for the latest NLME in Japan, we denoted our entire analysis pipeline with our prompts with model's output list as supplementary table to ensure the reproducibility. Moreover, we analyzed incorrect responses and associated explanations. These were certainly the novelty and different points from previous studies. We added the Kasai's paper and added some sentences mentioned above in our manuscript.

**Manuscript change(s) (bold):**

(Line in 112-115 in Introduction)

> *"Furthermore, the performance of the current GPT model (GPT-4) employing an estimated 10 trillion parameters [11] has not yet been* ***fully*** *evaluated on the latest Medical Licensing Examination, which was originally written in non-English texts and*

*held after the completion of GPT-4 model training (August 2022) [17].”*

(Line 212-217 in Discussion)

***“Although, a previous study showed that GPT-3.5 failed to achieve the minimum passing rates [21], Kasai, et al. first reported the potential performance of the GPT-4 for passing the NLMEs in Japan [22]. Our study, in addition to these previous reports, demonstrated that GPT-4 can pass the 117th NMLE with a higher percentage of correct answers than reported using our optimized prompts proposed herein.”***

**-Minor comments-**

**1.** *There were variations of “GPT3.5” and “GPT-3.5”.*

**Author reply:** Thank you for your advice. We corrected the variations.

**2.** *Some of the double quotes are "" instead of `` '' (LaTeX). Please check all the occurrences.*

**Author reply:** We corrected the double quotes.

**3.** *The term "Prompt tuning" has a certain meaning (learning prompt embedding through fine-tuning) in the machine learning literature, so if the authors just intended to use the created dataset for finding a prompt suitable for this task, a different name would better fit.*

**Author reply:** We appreciate your suggestion. We have changed all the phrases, “prompt tuning”, to “prompt optimization” or adjustment as below.

**Manuscript changes (bold):**

(Line 46 in Abstract)

*“We initially used **the GPT models and several prompts for** 290 questions…”*

(Line 48-9 in Abstract)

*“Thereafter, we tested the performance of the best GPT model (GPT-4) with **optimized** prompts on….”*

(Line 50-1 in Abstract)

*“The best model with the **optimized** prompts scored 82.7% ...”*

(Line 56 in Abstract)

*In conclusion, GPT-4 with our **optimized** prompts achieved…*

(Line 70-71 in Author summary)

*"Initially, we used an **optimization** dataset of 290 questions from the 116th NMLE, and then the GPT-4 model with **optimized** prompts was tested on 262 questions from the 117th NMLE."*

(Line 119-20 in Introduction)

*"…were used as a model and prompt performance **optimization** set before using the latest questions. …"*

(Line 127 in Results)

*"Improving performance through English translation and **optimized** prompts in 116th NMLE (2022)"*

(Line 139-40 in Results)

*"To further improve the correct answer rate and reduce the errors, we **adjusted** our prompts for each question type…"*

(Line 142 in Results)

*"These **optimized** prompts improved the correct answer rate to…"*

(Line 144 in Results)

*"Furthermore, we applied the above-**optimized** prompts to the GPT-4, which…*

(Line 148 in Results)

*"GPT-4 performance on 117th (2023) NMLE with **optimized** prompt*

(Line 149-50 in Results)

*"Thereafter, we evaluated that the performance of the best model (GPT-4) with **optimized** prompts for…"*

(Line 151 in Results)

*"With **optimized** prompts, the best model achieved a correct answer rate of…"*

(Line 154-5 in Results)

*"The present results were compared with the actual minimal passing rate on the examination. The current model with* ***optimized*** *prompts scored 82.7% (129/156) …"*

(Line 157-8 in Results)

*"Notably, we applied the GPT-4 model with* ***optimized*** *prompts to the entire set of 395 questions (text-only)"*

(Line 164-5 in Results)

*"To further enhance the performance of the model, we performed … by the optimal GPT-4 model with* ***optimized*** *prompts for the 117th NMLE questions."*

(Line 182-3 in Discussion)

*"The results indicate that 1) GPT-4 with* ***optimized*** *prompts cleared… 2) GPT-4 with* ***optimized*** *prompts also qualified the minimum passing rate on the latest 117th NMLE (2023) …"*

(Line 188 in Discussion)

*"Despite the absence of image data in the questions, this study demonstrated the first attempt to use the best available GPT-4 model with* ***optimized*** *prompts to achieve a minimum passing rate for the latest 117th NMLE in Japan."*

(Line 189 in Discussion)

*"First, GPT-4 with* ***optimized*** *prompts cleared the minimal passing rate on the 116th NMLE in Japan held in February 2022."*

(Line 196 in Discussion)

*"In particular, after* ***adjusting*** *our prompts to include a translation procedure into plain English and modifying the output format based on the question type, …"*

(Line 201-2 in Discussion)

*"Although the error rate increased to 14.8% upon translating the Japanese questions into English, it notably decreased to 7.6% after* ***adjusting*** *the prompts…"*

(Line 210-11 in Discussion)

*"Second, even in case of the latest 117th NMLE (2023), GPT-4 with* ***optimized*** *prompt*

*qualified the actual minimum passing rate."*

(Line 217-18 in Discussion)

*"The current results can be derived from the exquisite combination of essential factors such as English translation and optimally **adjusted** prompts for…"*

(Line 220-222 in Discussion)

*"Third, inadequate medical knowledge, …by the best available GPT model with **optimized** prompts."*

(Line 250-2 in Discussion)

*"The novelty of this study is that it is the first research to achieve … with the optimally **adjusted** prompts."*

(Line 252-54 in Discussion)

*"First, we only used questions without image data to evaluate the performance of the best available model with **optimized** prompts, although…"*

(Line 267 in Discussion)

*"In conclusion, GPT-4 with optimally **adjusted** prompts achieved…"*

(Line 278-80 in Materials and methods)

*"Initially, the questions from the 116th NMLE in Japan (February 2022) were used as a model and prompt **optimization** set to optimize …"*

(Line 280-2 in Materials and methods)

*"Subsequently, we assessed the performance of the best GPT model (GPT-4) with the **optimized** prompts for …"*

(Line 290-1 in Materials and methods)

*"In addition, all image-containing questions were removed from both the **prompt-optimization** and …"*

(Line 327-9 in Materials and methods)

*"We further refined the prompts using the 116th NMLE questions to achieve …, because prompt **fine-adjustment** can improve…"*

(Line 345-47 in Materials and methods)

*"We used the GPT-3.5 version GPT3.5-turbo-0301 for the "Japanese", "English", and "English with **optimized** prompts" analyses, and the GPT-4 model version released on March 14th 2023 for the "English with **optimized** prompts" analysis."*

(Line 360-2 in Materials and methods)

*"In the primary performance evaluation, we assessed the correct answer rate for questions without images in the 117th NMLE in Japan using the best GPT model (GPT-4) with **optimized** prompts, which was compared to the actual minimally passing rate on the examination."*

(Line 492-98 in Figure 1)

*"Questions from the 116th NMLE in Japan were used as the prompt **optimization** dataset and those from 117th NMLE were utilized as the performance-testing dataset after removing the image-based questions. During the prompt **optimization** process, questions from the prompt **optimization** dataset were input into GPT-3.5-turbo and GPT-4, using simple prompts in both Japanese and English along with **optimized** prompts in English. Subsequently, we evaluated the outputs from GPT-3.5-turbo and GPT-4 with **optimized** prompts. After **adjusting** the prompts, the GPT-4 model with the **optimized** prompts was tested on the performance-testing dataset (117th NMLE)."*

(Line 500-5 in Figure 2)

*"Figure 2. Variations in the rate of correct answers across languages, prompt **adjusting** levels, and GPT models.*

*Translating the Japanese questions into English text improved the correct answer rate; however, it increased the output error rate. Upon further **adjusting** the prompts, the correct answer rate improved and the output error decreased. Moreover, switching from the GPT-3.5 model to the GPT-4 model enhanced the correct answer rate and almost eliminated errors."*

(Line 511 in Figure 3)

*"C: Our optimized "English with **optimized** prompts"."*

(Table.1 in Tables)

*"Table 1. Performance of optimal GPT-4 model with **optimized** prompt for the 117th*

*NMLE in Japan.”*

**4.** *I strongly recommend the authors to check the source codes available at GitHub by using a lint tool such as flake8, pep8, etc.*

**Author reply:** Thank you for your advice. We again arranged the source codes at GitHub.

**Response to Reviewer #2**

**-Major comments-**

**1.** *GPT-3.5, ChatGPT, and GPT-4 are different models. Each of the language models you used in the experiments should be clearly described. Eg., "GPT-4 powered ChatGPT" (lines 55, 74 and 261) should be "GPT-4", "ChatGPT with GPT-3.5 and GPT-4" should simply be "GPT-3.5 and GPT-4", and so on. There are lots of such mentions.*

**Author reply:** We apologize for your confusion. As you pointed out, GPT-3.5 and GPT-4 are models, and ChatGPT is an application of these models. We corrected our unappreciated expressions.

**Manuscript changes (bold):**

(Line 40 in Abstract)

> *"Although **GPT-3.5** recently scored high on …"*

(Line 43 in Abstract)

> *"This study assessed **GPT's** performance on…"*

(Line 49 in Abstract)

> *"Thereafter, we tested the performance of the best **GPT** model (GPT-4) with …"*

(Line 69-70 in Author summary)

> *"This study assessed the performance of **GPT-3.5 and GPT-4** models in Japan's National Medical Licensing Examination (NMLE)."*

(Line 105-7 in Introduction)

> *"Although GPT is a non-domain-specific LLM, not exclusively intended to be used for medical or healthcare fields, recent publications have demonstrated that **GPT-3.5** possesses sufficient ability to …"*

(Line 108-9 in Introduction)

> *"In contrast, another study reported **GPT-3.5's** inadequate performance on non-English-based Korean medical questions [16]."*

(Line 109-12 in Introduction)

> *"Although the performance variation can be attributed to …, the relationship between*

*these differences and **GPT's** performance in answering medical questions remains unclear."*

(Line 113-14 in Introduction)

*"Furthermore, the performance of **the current GPT model (GPT-4)** employing an estimated 10 trillion parameters [11] has not yet been fully evaluated on …"*

(Line 128-9 in Results)

*"Initially, we used the non-image-based questions from 116th NMLE in Japan to develop the optimal input prompts for **GPT** to maximize the correct answer rate."*

(Line 133-4 in Results)

*"Using the ChatGPT API powered by **GPT-3.5**, we initially tested its performance for the original questions in Japanese language."*

(Line 135-6 in Results)

*"Accordingly, we used updated prompts to translate the original Japanese NMLE questions into English using **GPT-3.5** before inputting them as questions."*

(Line 144 in Results)

*"Furthermore, we applied the above-optimized prompts to the **GPT-4**, which demonstrated …"*

(Line 148 in Results)

*"**GPT-4** performance on 117$^{th}$ (2023) NMLE with optimized prompt"*

(Line 163 in Results)

*"Exploratory analysis of incorrect **GPT-4** responses and their associated explanations"*

(Line 171-2 in Results)

*"In terms of Japan-specific medical system, **GPT-4** failed to adequately answer questions related to …"*

(Line 186-7 in Results)

*"Despite the absence of image data in the questions, this study demonstrated the first attempt to use the best available **GPT-4** model with …"*

(Line 190-1 in Results)

*"Although **GPT-3.5** achieved a correct answer rate of 52.8% for Japanese questions, it increased to 56.2% after translating the questions into English."*

(Line 206-7 in Results)

*"Finally, upon applying these optimized prompts to **GPT-4**, the correct response rate increased to 82.8% and the error rate plummeted to 1.0%."*

(Line 212-3 in Results)

*"Although, a previous study reported that **GPT-3.5** failed to achieve the minimum passing rates [21], …"*

(Line 217-9 in Results)

*"The current results can be derived from the exquisite combination of essential factors such as English translation and optimally adjusted prompts for obtaining correct answers through the best performance of the latest **GPT** model."*

(Line 220-2 in Results)

*"Third, inadequate medical knowledge, information related to the medical and healthcare system guidelines of Japan, and mathematical errors formed the three major factors of the incorrect answers generated by the best available **GPT** model …"*

(Line 231-2 in Results)

*"Although the **GPT-4** delivered improved performance in terms of output differences between the languages, …"*

(Line 235-6 in Results)

*"Moreover, an instruction of "approximating the decimal place" was not properly comprehended by **GPT-4** during the Japanese-to-English translation."*

(Line 238-9 in Results)

*"…, indicating that calculation problems may be a relatively unsuitable field for the current **GPT** model."*

(Line 240-1 in Results)

*"As discussed, we express strong concerns regarding the use of the current **GPT** for medical purposes,"*

(Line 250-1 in Results)

*"The novelty of this study is that it is the first research to achieve a minimum passing rate using 262 non-image questions in the latest 117$^{th}$ NMLE in Japan with the **GPT-4** version with ..."*

(Line 267-8 in Results)

*"In conclusion, **GPT-4** with optimally adjusted prompts achieved a minimum passing rate in the latest 117th NMLE in Japan."*

(Line 315-6 in Materials and methods)

*"In this study, we used both **the GPT-3.5 and the GPT-4 versions**."*

(Line 319-20 in Materials and methods)

*"We used the 116$^{th}$ NMLE in Japan to generate the most suitable prompts for **GPT** to answer the 117$^{th}$ NMLE questions."*

(Line 320-1 in Materials and methods)

*"Using the ChatGPT API, we first instructed **GPT** to respond to the original questions in Japanese language."*

(Line 323-4 in Materials and methods)

*"Second, we instructed **GPT** to translate the original Japanese NMLE questions into English using its own capabilities before inputting them as questions (Figure 3B)."*

(Line 327 in Materials and methods)

*"Finally, we inquired **GPT** to improve the prompt itself."*

(Line 334-5 in Materials and methods)

*"In brief, **GPT** was initially instructed to translate the HTML-based Japanese questions into ..."*

(Line 343-5 in Materials and methods)

*"Specifically, eight investigators (Y. T., T. N., K. A., T. E., R. M., S. K., H. K., and F. H)*

*inputted the questions, choices, and appropriate prompts into* ***GPT*** *and summarized the output answers."*

(Line 350-1 in Materials and methods)

*"We manually compared* ***GPT's*** *output answers with the official answers to determine the correctness of the output answers."*

(Line 360-1 in Materials and methods)

*"In the primary performance evaluation, we assessed the correct answer rate for questions without images in the 117th NMLE in Japan using the best* ***GPT*** *model (GPT-4) with optimized prompt, ..."*

(Line 366-8 in Materials and methods)

*"Furthermore, we analyzed the content of the incorrect answers along with their explanations to identify the areas in which the application of the current* ***GPT*** *for medicine may be relatively weak."*

(Line 374-5 in Acknowledgments)

*"We also thank* ***GPT-4*** *and Enago English proofreading service for English proofreading."*

(Line 497-8 in Figure 1)

*"After adjusting the prompts, the* ***GPT-4*** *model optimized with the adapted prompts was tested on the performance-testing dataset (117th NMLE)."*

(Line 513-4 in Figure 3)

*"****GPT*** *was initially instructed to translate HTML-based Japanese questions into simple, direct, and improved English."*

(Line 530-3 in Figure 4)

*"The long-term treatment goal for diabetes is strict blood sugar control, but in this case, strict blood sugar control with sulfonylurea drugs during the initial treatment may aggravate the risk of diabetic retinopathy, raising strong concerns on* ***GPT-4*** *answer."*

(Line 534-6 in Figure 4)

*"Note: We re-inputted the prompts to obtain the above figures, and the text-contexts of*

*the explanations from **GPT-4** differed slightly from that ...”*

**2.** *Line 96: BERT is a masked language model to obtain token representations in context, which is dis-similar to other generative language models such as LaMDA, PaLM, LLaMA and GPT models.*

**Author reply:** I appreciate your suggestion. We removed the BERT from the sentence.

**Manuscript change(s) (Line 96-99 in Introduction):**

> *“A few notable LLMs include Language Models for Dialog Applications (LaMDA) [6], Pathway Language Model (PaLM) [7], Large Language Model Meta (LLaMA) [8], and Generative Pretrained Transformer (GPT-3) and later models [9-11].”*

**3.** *Lines 116 and 151: GPT-4 was announced in March 2023, and there is no GPT-4 model trained in August 2022.*

**Author reply:** Thank you for your comment. As you pointed out, GPT-4 was announced in March 2023. However, according to the GPT-4 Technical Report [12] (page 42, line 6), the GPT-4 model had completed its training in August 2022. The six-month interval has been spent for evaluating, testing, and iteratively improving the model and the system-level.

**4.** *Line 135: What does "output error" mean? There is no explanation about the difference between incorrect answers and output errors. Worth than that, line 351 says "We define the output errors as incorrect answers", but they should be different as Figure 2 shows them differently.*

**Author reply:** I appreciate your suggestion. We defined an "output error" as a case where a question was not answered, or the number of answers was incorrect. We have added this explanation in Materials and methods.

**Manuscript change(s) (Line 353-354 in Materials and methods) (bold):**

> ***“We defined output errors as a case where a question was not answered, or the number of answers was incorrect. These output errors were handled as wrong answers.”***

**5.** *There are a number of papers that investigated the ability of ChatGPT and GPT-4 by applying them to various problems. This paper is one of such an attempt. The originl part of this paper is the method for designing the prompts to tune the LLMs to solve the Japanese medical exams.*

*While examples are shown in appendix, no detail of the ways to obtain the optimal prompts is described. It is good if this method can be generalized. However, if this is done in a heuristical way by hand, the readers cannot learn from this research.*

**Author reply:** We appreciate your critical comments. In this study, we compare three approaches. First, ChatGPT was asked to solve the questions as the original text in Japanese. Second, the questions were translated into English. Finally, the questions were translated into simple English and the prompts were structured to provide detailed instructions on how to answer the questions with the specific sample output style. By comparing these three processes, the translation into plain English and the structuring of the prompts succeeded in eliciting the desired output, resulting in a lower error rate and a higher percentage of correct answers. Our structured prompts were not automatically generated with the certain algorithm. Therefore, we added sentences in the limitation paragraph in the Discussion.

**Manuscript change(s) (line262-266 in Discussion) (bold):**

> ***"Finally, it should be noted that our adapted prompts were not automatically generated with a specific algorithm. However, our step-wise approach surely improved the performance of answering questions of the NMLE in Japan, that might support the evidence that adapted structured prompts could be one of the options for maximizing scoring performance."***
