# Supplementary material for: Performance of Generative Pretrained Transformer on the National Medical Licensing Examination in Japan
Source: PLOS Digit Health. 2024 Jan 23;3(1):e0000433. doi: 10.1371/journal.pdig.0000433 (PMC10805303; doi:10.1371/journal.pdig.0000433)
Supplement: Attachment — Submitted filename: Response to Reviewers.docx [file pdig.0000433.s003.docx]

**Response to Reviewers for PDIG-D-23-00146R2**

**“Performance of Generative Pretrained Transformer on the National Medical Licensing Examination in Japan”**

We once again thank to the editor and reviewers for their constructive comments during this subsequent review round. Detail responses are provided below.

**Response to editor:**

1. Please review your reference list to ensure that it is complete and correct. If you have cited papers that have been retracted, please include the rationale for doing so in the manuscript text, or remove these references and replace them with relevant current references. Any changes to the reference list should be mentioned in the rebuttal letter that accompanies your revised manuscript. If you need to cite a retracted article, indicate the article's retracted status in the References list and also include a citation and full reference for the retraction notice.

**Author reply:** We again reviewed our reference list and confirmed it was correct.

2. Please send a completed 'Competing Interests' statement, including any COIs declared by your co-authors. If you have no competing interests to declare, please state "The authors have declared that no competing interests exist". Otherwise please declare all competing interests beginning with the statement "I have read the journal's policy and the authors of this manuscript have the following competing interests:"

3. If you did not receive any funding for this study, please simply state: “The authors received no specific funding for this work.”

**Author reply:** Thank you for the comments. We added the COI and Funding sections in the manuscript.

**Manuscript changes (Bold) (Competing interest):**

***“Competing interest***
 ***The authors have declared that no competing interests exist.***

***Funding
 None.”***

4. We ask that a manuscript source file is provided at Revision. Please upload your manuscript file as a .doc, .docx, .rtf or .tex.

5. Please provide separate figure files in .tif or .eps format only and remove any figures embedded in your manuscript file. Please also ensure that all files are under our size limit of 10MB.

**Author reply:** We have uploaded our manuscript file as a .docx format. We have also provided separate figure files in .tif format.

6. Please update the source code in Github to help the community.

**Author reply:** We have updated our revised source code in Github (https://github.com/yudaitanaka1026/ChatGPT_NMLE_Japan/).

**Response to Reviewer #1:**

**1.** *In the response letter, you wrote "However, since they have not shown any detailed prompts they used for the analyses in the preprint manuscript (even for their version 2 paper), we could not replicate or confirmed validity of any of their results." but you can find the prompts at their GitHub repository (https://github.com/jungokasai/IgakuQA/blob/main/scripts/prompts/prompt.jsonl), which was clearly mentioned from their paper.*

**Author reply:** Thank you for providing the information regarding the prompts. We apologize for the oversight related to the GitHub repository webpage mentioned in the footnote of the preprint paper. We could find the specific prompts the authors used in their study.

**2.** *Also, I recommended you to check your Python code at GitHub, and you wrote "Thank you for your advice. We again arranged the source codes at GitHub.", but the source code was not updated at all, so I withhold recommending the paper as a full acceptance (you wrote you arranged your code on GitHub, but it is not true. Maybe you forgot to push?). https://github.com/yudaitanaka1026/ChatGPT_NMLE_Japan*

**Author reply:** Thank you for drawing attention to the issue with our GitHub code. We have re-checked and subsequently updated our code on GitHub (https://github.com/yudaitanaka1026/ChatGPT_NMLE_Japan/). Please inform us of any further concerns.
