# Supplementary material for: Performance of Generative Pretrained Transformer on the National Medical Licensing Examination in Japan
Source: PLOS Digit Health. 2024 Jan 23;3(1):e0000433. doi: 10.1371/journal.pdig.0000433 (PMC10805303; doi:10.1371/journal.pdig.0000433)
Supplement: Attachment — Submitted filename: 231217_Rev3 Response to Reviewers_CLEAN.docx [file pdig.0000433.s004.docx]

**Response to Reviewers for PONE-D-23-04728R3**

**“Performance of Generative Pretrained Transformer on the National Medical Licensing Examination in Japan”**

We very thank to the editor and reviewers for their comments during this subsequent review round. Detail responses are provided below.

**Response to Reviewer #1**

**1.** *I can hardly find where you treat the Kasai et al.'s work adequately. If you think the optimization of the prompts is the contribution, you need to compare your prompt against theirs, but Table 1 contains only the results with the optimized prompt (I cannot find the baseline results for example with Kasai et al.'s prompt, nor a baseline result without your optimization).*

**Author reply:** Thank you very much for your productive comments. According to your suggestion, we directly compare the prompt performance from the Kasai et al.’s GitHub repository data (available results up to 2022 [116^th^] data: https://github.com/jungokasai/IgakuQA) regarding prompt optimization to highlight our research. We compared our results to theirs at our prompt optimization phase, and we found that our prompt optimized model showed higher scoring rates than those using their state-of-the art [SoA] model prompt. Also, we clarified our baseline results without our optimization in the Figure 2 (focusing on the results of prompt optimization phase). Aligned with above corrections, we integrated the rates as scoring rates (e.g., we corrected all the word, “a minimum passing rate”, to “a minimum passing ***scoring*** rate”) and recalculated all of them in this manuscript. Furthermore, we corrected the sentence in the Discussion regarding the assessment of 117^th^ NMLE results with previous studies.

**Manuscript change(s) (bold):**

(Abstract)

*“… The best model with the optimized prompts scored 82.7% for the essential questions and 77.2% for the basic and clinical questions, both of which sufficed* ***the minimum passing scoring rates*** *of 80.0% and 74.6%, respectively. After an exploratory analysis of 56 incorrect answers from the model, we identified the three major factors contributing to the generation of the incorrect answers—insufficient medical knowledge, information on Japan-specific medical system and guidelines, and mathematical errors. In conclusion, GPT-4 with our optimized prompts achieved* ***a minimum passing scoring rate*** *in the latest 117^th^ NMLE in Japan. …”*

(Author summary)

*“… Initially, we used an optimization dataset of 290 questions from the 116th NMLE, and then the GPT-4 model with optimized prompts was tested on 262 questions from the 117th NMLE. The model scored 82.7% for essential and 77.2% for basic and clinical questions, surpassing* ***the minimum passing scoring rates.*** *Incorrect answers were attributed to insufficient medical knowledge, Japan-specific medical system information, and mathematical errors. In conclusion, GPT-4 achieved* ***a minimum passing scoring rate*** *and can be considered a valuable tool for fulfilling the needs of medical and healthcare fields.”*

(Line in 128-129 in Results)

*“Initially, we used the non-image-based questions from 116th NMLE in Japan to develop the optimal input prompts for GPT to maximize* ***the scoring rates.****”*

(Line in 134-136 in Results)

*“Initially, we obtained the* ***scoring rates of 53.7 % (88/164 points) for essential questions and 52.4% (110/210 points) for basic and clinical questions*** *with an output error rate of 5.5%.”*

(Line in 137-139 in Results)

*“Although this marginally increased* ***the scoring rates to 60.4 % (99/164) for essential questions and 54.8% (115/210) for basic and clinical questions****, the output errors increased to 14.8% (Figure 2).”*

(Line in 140-141 in Results)

*“To further improve* ***the scoring rates*** *and reduce the errors, we adjusted our prompts for each question type (Basics of Medicine, Clinical Medicine, and Comprehension).”*

(Line in 143-145 in Results)

*“These optimized prompts improved* ***the scoring rates to 64.6 % (106/164) for essential questions and 62.9% (132/210) for basic and clinical questions*** *with a reduced output error rate of 7.6% (22/290).* *Additionally, we applied the above-optimized prompts to the GPT-4, which demonstrated* ***the scoring rates of 90.9 % (149/164) for essential questions and 81.4% (171/210) for basic and clinical questions*** *and a minimal error rate of 1.0% (3/290) (Figure 2).”*

(Line in 148-153 in Results)

*“****Furthermore, we applied the GPT-4 model with optimized prompts to the entire set of 393 questions (text-only) in the 116th NMLE. This optimal model also showed a scoring rate of 88.7% (173/195) for essential questions and 78.5% (233/297) for basic and clinical questions, whose rates were higher than the minimum passing scoring rates as well as those previously reported state-of-the-art model [18].****”*

(Line 158-161 in Results)

*“With optimized prompts, the best model achieved* ***a scoring rate of 82.7 % (129/156) for essential questions and 77.2% (139/180) for basic and clinical questions, both of which qualified the minimum passing scoring rates of 80.0% and 74.6%, respectively (Figure 2) [19], and an output error rate of 0.8% (Table 1).****”*

(Line 194-197 in Discussion)

*“Although GPT-3.5 achieved* ***Although GPT-3.5 achieved a scoring rate of 53.7% for essential questions and 52.4% for basic and clinical questions*** *for Japanese questions, it increased to* ***60.4% for essential questions and 54.8% for basic and clinical questions*** *after translating the questions into English.”*

(Line 201-204 in Discussion)

*“In particular, after adjusting our prompts to include a translation procedure into plain English and modifying the output format based on the question type,* ***the scoring rates increased to 64.6% for essential questions and 62.9% for basic and clinical questions.****”*

(Line 212-215 in Discussion)

*“Finally, upon applying these optimized prompts to GPT-4,* ***the scoring rates increased to 90.9% for essential questions and 81.4% for basic and clinical questions with higher scoring rates than the previously reported state-of-the-art model prompt,*** *and the error rate plummeted to 1.0%.”*

(Line 221-223 in Discussion)

*“Our study, in addition to these previous reports, demonstrated that GPT-4 can* ***also surpass the minimum passing scoring rate of*** *the latest 117th NMLE in Japan using our optimized prompts proposed herein.”*

(Line 325 in Materials and methods)

*“Prompt engineering to maximize* ***the scoring rates****”*

(Line 335 in Materials and methods)

*“The target outcome of this study is* ***the scoring rates.****”*

(Line 357-367 in Materials and methods)

*“****In addition, the scoring rate was evaluated by giving scoring weights to the questions: 1 point for basic medicine questions and 3 points for clinical medicine and comprehension questions, following the scoring criteria of the MHLW, Japan.*** *We defined output errors as a case where a question was not answered, or the number of answers was incorrect. These output errors were handled as wrong answers.* ***To further evaluate performance of the optimized prompt, we compared scoring results obtained with our optimized prompt with those achieved using the state-of-the-art model prompt, as reported in the previous paper [18] during the prompt optimization phase.”***

(Line 370-371 in Materials and methods)

*“In the primary performance evaluation, we assessed* ***the scoring rates*** *for questions without images in the 117th NMLE in Japan using the best GPT model (GPT-4) with optimized prompts, which was compared to the actual* ***minimally passing scoring rate*** *on the examination.…”*

(Line 372-374 in Materials and methods)

*“In the secondary performance evaluation, we examined* ***the scoring rates*** *for all questions in the 117th exam using the optimal model and prompts.”*

(Line 514-519 in Figure legends)

*“Figure 2. Variations in the* ***scoring rates*** *across languages, prompt adjusting levels, and GPT models.*

*Translating the Japanese questions into English text improved the* ***scoring rates****; however, it increased the output error rate. Upon further adjusting the prompts, the* ***scoring rates*** *improved, and the output error decreased. Moreover, switching from the GPT-3.5 model to the GPT-4 model enhanced the* ***scoring rates*** *and almost eliminated errors.”*

(Figure 2)

(Table 1) (highlighted)

**2.** *Also, I thank you to check that the GitHub repository is correctly updated, but I can find that the update is only for fixing typos. I specifically asked you to use some style-checking tools like flake8, and you said you did so, but there seems no indication that you did so. The source code seems like a script that can be run with Google Colab, but I strongly recommend you to use some lint tools to check the source code.*

**Author reply:** We appreciate your suggestion to use a lint tool such as flake8 to enhance our code quality. Following your advice, we have conducted a thorough check of our code using flake8 and have updated the improved version on our GitHub repository.
(https://github.com/yudaitanaka1026/ChatGPT_NMLE_Japan/)
